# Spontaneous, Non-Traumatic Retropharyngeal Abscess Complicated by Spinal Osteomyelitis and Epidural Abscess in Immunocompetent Adult: Management and Proposal Pathophysiological Mechanism

**DOI:** 10.7759/cureus.9028

**Published:** 2020-07-06

**Authors:** Giorgos Sideris, Thomas Nikolopoulos, Nikolaos Papadimitriou

**Affiliations:** 1 2nd ENT Department, Attikon University Hospital, National and Kapodistrian University of Athens, Athens, GRC

**Keywords:** epidural abscess, infection, osteomyelitis, retropharyngeal abscess

## Abstract

Retropharyngeal abscess (RPA) in adults is a potentially life-threatening condition that relates in most of the cases with local trauma. Non-traumatic RPA complicated by spinal osteomyelitis and epidural abscess is a rare entity in immunocompetent adults and represents an emergency medical condition when the patient develops neurologic symptoms. This article presents a case of non-traumatic RPA complicated by spinal osteomyelitis and epidural abscess in a 77-year-old male with the free past medical history. We highlight the importance of early and meticulous daily drainage as well as sequential MRI scanning for early diagnosis, treatment and follow-up for signs of vertebral involvement. The patient had a full recovery and was subsequently discharged with per os four-month antibiotic treatment. The definitive mechanism is unclear for non-traumatic cases with “hematogenous path” being the closest description. Spontaneous RPA can develop from bacteria infection spread of adjacent structures, local inflammatory process and microthrombosis formation can impair the blood supply of vertebral and intervertebral disks.

## Introduction

Retropharyngeal abscess (RPA) is usually observed in children younger than five-year-old [1]. RPA in adults is a rare and potentially life-threatening medical condition that relates to local trauma from ingested foreign bodies in 60% of the cases [2]. Vertebral osteomyelitis is an uncommon and serious medical condition that can develop after systemic or iatrogenic infections and local trauma [3]. Spinal involvement of RPA can cause irreparable neurologic damages or sometimes death if not recognised and treated early [4].

## Case presentation

Α 77-year-old male presented at the emergency department of a provincial hospital complaining of dysphagia, hoarseness, neck and lumbar pain. He had chills and fever up to 39°C. His laboratory tests revealed white blood cell count: 12.360 Κ/μL and C-reactive protein: 80.8 mg/L. His past medical history was free. He denied any trauma or foreign body ingestion. Clinical examination was unremarkable and chest X-ray had no findings. There was no breathing impairment or palpable neck masses. He was admitted and treated under broad-spectrum antibiotics.

A CT scan was performed that revealed an RPA that was drained intraorally under local anaesthesia.

The following days his symptoms had partially improved but neck and lumbar pain persisted. The patient was still febrile and it was decided to transport him to our department after 10 days of hospitalisation. Clinical examination revealed pyorrhoea from the posterior pharyngeal wall. Daily drainage was performed with no improvement and it was decided to resume imaging tests.

**Figure 1:**
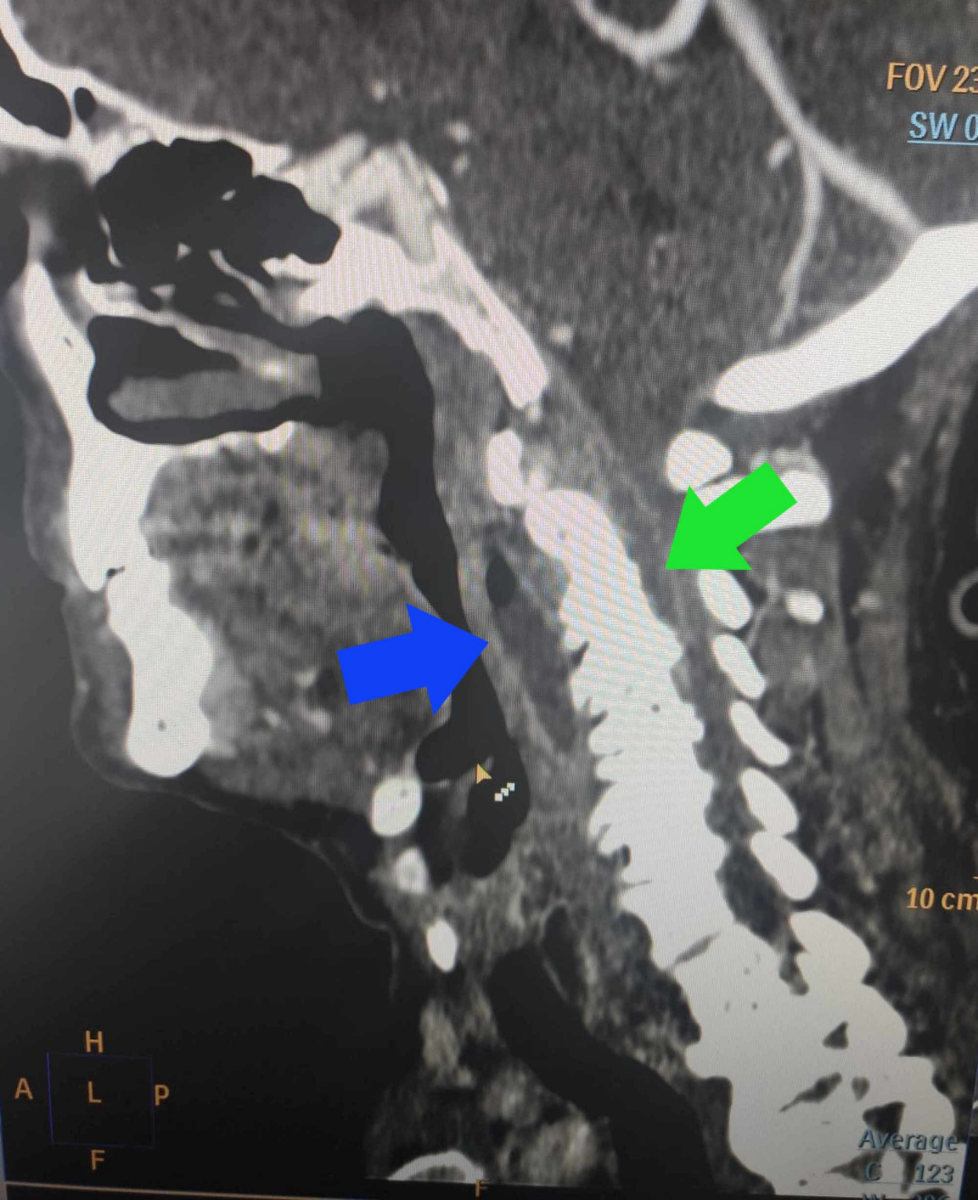
CT scan Contrast-enhanced computerized tomography scan with existence of retropharyngeal abscess (blue arrow) and epidural abscess (green arrow)

CT scan revealed RPA with an epidural abscess in the cervical segment of the spine, distorted cervical vertebral anatomy and thrombosis of the left jugular vein (Figure 1).

**Figure 2:**
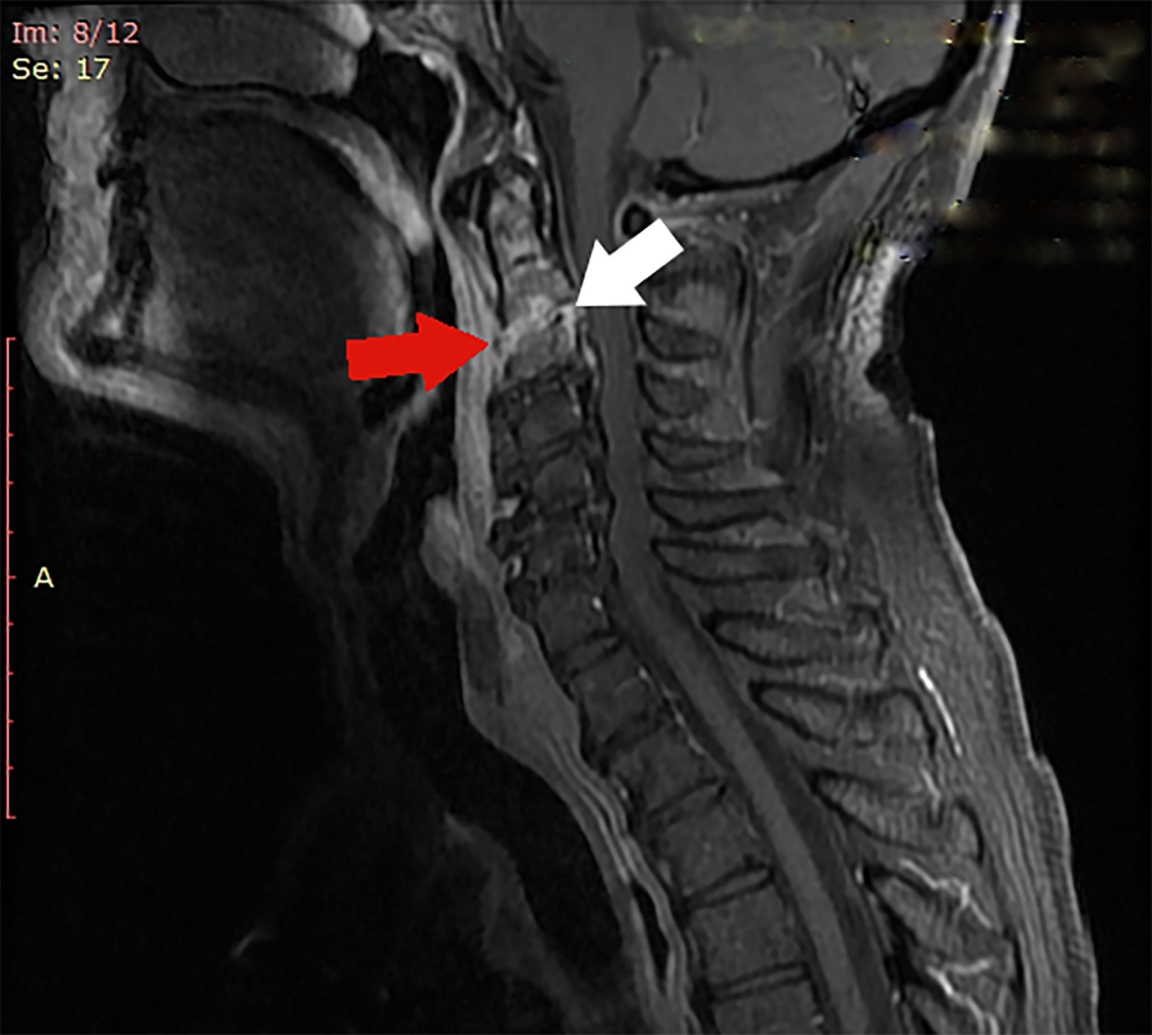
MRI scan MRI that shows C2/3 spondylodiscitis (red arrow) and epidural abscess with cord compression (white arrow)

MRI scan revealed cervical osteomyelitis involving the second and third vertebral bodies, prevertebral and epidural abscess with spinal cord compression. Spinal stenosis in C5/6 segment was also observed (Figure 2).

The possibility of injury from foreign body and tuberculosis was excluded by clinical examination, imaging and lab tests.

Neurosurgical and orthopaedic assessment recommended conservative treatment as far as there was no neurological involvement.

**Figure 3:**
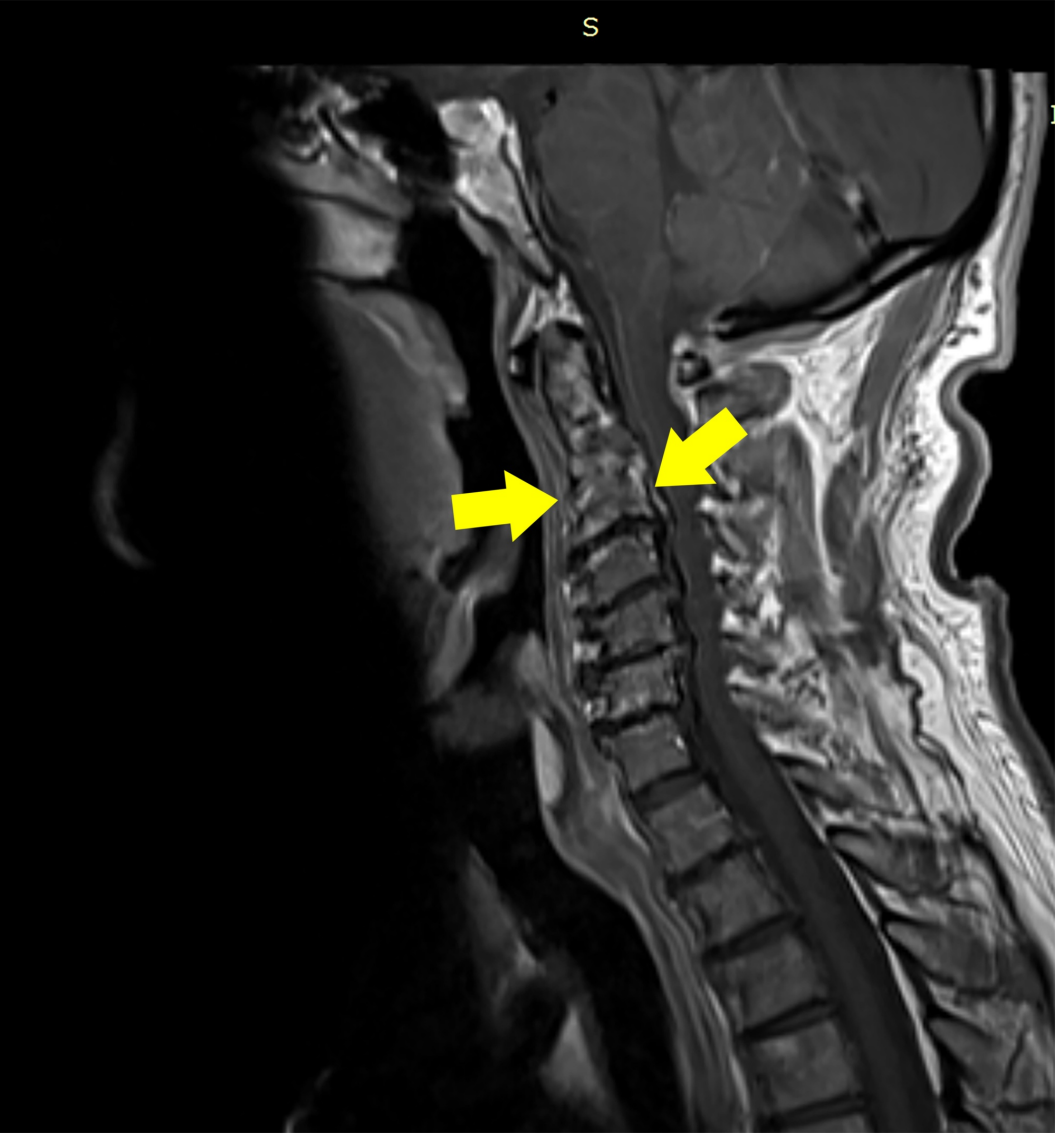
Follow-up MRI scan Four-month follow-up MRI showing resolution of retropharyngeal and epidural abscess (yellow arrows)

The patient continued his treatment with antibiotics, low molecular-weight heparin (LMWH), analgesia and hydration. Instructions for using soft cervical collar and bed rest were given. Drainage of the RPA was performed daily under local anaesthesia. Pus was sent for cultures that were positive for Staphylococcus aureus. Follow-up imaging with MRI and laboratory tests showed improvement (Figure 3).

He was discharged on day-42, after six weeks of hospitalisation and combination of IV and oral antibiotics for four months.

## Discussion

RPA occurs most frequently in children younger than six years old, due to retropharyngeal space lymph node degeneration after adolescence. In adults, cases have been reported in patients with immunodeficiency such as diabetes mellitus, HIV infection, patients undergoing hemodialysis or related to trauma and foreign bodies [2,5]. Our patient was immunocompetent with free past medical history for diabetes mellitus, HIV, haematological diseases, trauma and foreign bodies.

Early diagnosis of RPA is crucial as it can affect neighbouring structures and can lead to airway obstruction, sepsis, suppurative mediastinitis, aspiration pneumonia, empyema or carotid artery erosion [6]. Thrombosis of the jugular vein as in our patient is the result of the infection and it was treated conservatively with LMWH.

The incidence of RPA associated with epidural space abscess is very rare [7].

A pathophysiological mechanism for trauma-related cases has been proposed [8].

In non-traumatic cases, the definitive mechanism is unclear. Spontaneous RPA can develop from bacteria infection spread of adjacent structures of upper aerodigestive track like tonsils, nasopharynx and sinuses. Local inflammatory process and microthrombosis formation can impair the blood supply of vertebral and intervertebral disks. Bone and disk ischemic necrosis and the direct action of bacteria proteolytic enzymes can lead to destruction, deformity and epidural abscess formation. Many authors have reported such cases as a result of bacteria “seeding” in the course of systemic infections originating from genitourinary tract, skin, soft tissues, respiratory tract, gastrointestinal tract, oral cavity and endocarditis [9]. Staphylococcus aureus is one of the predominant microorganisms in various reported cases [5].

Diagnostic tests to evaluate vertebral osteomyelitis include CT, contrast MRI and CT-guided biopsy from the vertebral lesion for cultures. Although CT scan is useful describing bone lesions and early destruction of the discs, MRI is the method of choice for diagnosis and follow-up with 96% sensitivity and 92% specificity. Furthermore, it is more accurate in the differential diagnosis of infectious lesions caused by neoplastic and degenerative lesions of the spine, identifying tissue inflammation and spinal cord compression [9].

Epidural abscesses in cervical spondylodiscitis can cause severe and rapidly worsening neurological symptoms [8]. Several authors have reported successful treatment with antibiotics [10]. Other authors have recommended surgical drainage, laminectomy, and debridement [2-4,7]. Indications for surgery are the failure of conservative treatment, progressive or residual neurological symptoms, and mechanical instability or spinal deformity. Furthermore, patient's age over 65 years old with diabetes, methicillin-resistant staphylococcus aureus (MRSA) infection or neurologic compromise are clearly recognized factors that increase the failure rate of the conservative treatment in spondylodiscitis with epidural abscess [11]. We confirmed literature recommendations for IV antibiotic therapy over the course of six to eight weeks and follow up under oral antibiotics for a total treatment duration of four months [12].

## Conclusions

RPA complicated by cervical spondylodiscitis and an epidural abscess is a rare entity. RPA can involve the cervical spine and disk segments in the development of the disease therefore, one should keep a high suspicious index. “Hematogenous path” is the closest description and a proposal pathophysiological mechanism for intraspinal involvement in non-traumatic RPA cases. Treatment requires daily drainage and IV antibiotics for several months. MRI scanning is recommended for early diagnosis and follow up. Bacterial cultures should define the choice of antibiotic treatment. Orthopaedic or neurosurgical intervention is needed when symptoms from cervical spine develop.
